# Paclitaxel retention of drug-coated balloons in veins and arteries under arteriovenous fistula hemodynamics

**DOI:** 10.3389/fcvm.2026.1888798

**Published:** 2026-10-07

**Authors:** Linda B. Liu, Kayla Lowry, Brystol Habermacher, Saami K. Yazdani

**Affiliations:** 1 Department of Biomedical Engineering, Wake Forest University School of Medicine, Winston-Salem, NC, United States; 2 Department of Engineering, Wake Forest University, Winston-Salem, NC, United States

**Keywords:** arteriovenous fistula, dialysis access, drug-coated balloon, *ex vivo* model, paclitaxel, pharmacokinetics, vascular bioreactor

## Abstract

**Introduction:**

Arteriovenous fistulas (AVFs) are the preferred vascular access for hemodialysis, but venous stenosis remains a major limitation to long-term patency. Drug-coated balloons (DCBs) have emerged as a promising therapy to inhibit neointimal hyperplasia and reduce restenosis. However, inconsistent clinical outcomes have limited their adoption for AVF stenosis. The unique AVF environment—characterized by high flow, turbulent shear stress, and alternating arterial and venous segments—may influence drug transfer and retention within the vessel wall. Despite its importance, the impact of tissue type under AVF-specific hemodynamics on DCB performance remains poorly understood. This study investigates paclitaxel retention in venous and arterial tissue under simulated AVF conditions using an *ex vivo* bioreactor system.

**Materials and methods:**

The IN.PACT™ AV paclitaxel-coated balloon was deployed for 2 min in porcine veins and arteries within an *ex vivo* AVF-simulating bioreactor. Treated vessel segments were collected at 1- and 24 h to quantify tissue paclitaxel concentration. Scanning electron microscopy (SEM) was performed at 1 h to assess coating deposition on the luminal surface.

**Results:**

Hemodynamic conditions were comparable between venous and arterial groups, confirming physiologically relevant AVF flow and pressure. On log-transformed tissue paclitaxel concentrations, no statistically significant difference was detected between tissue types at either time point (two-way ANOVA, *p* = 0.163; adjusted geometric mean ratio [vein/artery] = 2.69, 95% CI: 0.65–11.18), and drug levels decreased from 1- to 24 h in both groups. Venous samples exhibited greater variability, particularly at 1 h. SEM analysis revealed heterogeneous coating transfer in both tissues, with clustered deposits in veins and more distributed coverage in arteries.

**Conclusion:**

This study establishes an *ex vivo* platform for mechanistic evaluation of catheter-based drug delivery in vascular access. Under AVF hemodynamics, no statistically significant difference in acute paclitaxel retention was observed between venous and arterial tissues despite structural differences (log-scale two-way ANOVA, adjusted geometric mean ratio [vein/artery] = 2.69, 95% CI: 0.65–11.18). This absence of detectable differences suggests that tissue type alone does not drive DCB performance in AVFs.

## 1 Introduction

Arteriovenous fistulas (AVFs) are the preferred vascular access for hemodialysis due to their superior long-term patency and lower infection risk compared with grafts and catheters (1). Despite these advantages, AVFs are highly susceptible to failure, most commonly due to venous stenosis driven by neointimal hyperplasia. This pathology is influenced by a combination of surgical injury, abnormal shear stress, and turbulent flow conditions unique to AVFs (2).

Drug-coated balloons (DCBs), which deliver the antiproliferative agent paclitaxel to the vessel wall, have shown clear benefit in peripheral arterial disease by reducing restenosis and improving vessel patency. However, in the context of AVFs, clinical outcomes have been inconsistent, and current guidelines provide no recommendation for routine use of DCBs for AVF stenosis (3, 4). This discrepancy may suggest that the mechanisms governing drug delivery and retention in AVFs may differ fundamentally from those in peripheral arteries.

One key difference lies in the hemodynamic environment. AVFs are characterized by high flow rates, oscillatory shear stress, and the interaction of arterial and venous segments, all of which may influence drug transfer, tissue uptake, and retention. In addition, veins and arteries differ substantially in structure and composition. Veins exhibit greater permeability and compliance, whereas arteries have more organized elastin-rich matrices, which may affect the transport and binding of hydrophobic drugs such as paclitaxel. These competing factors make it unclear whether one tissue type is inherently more favorable for drug retention.

Although the pharmacokinetics of DCBs have been extensively studied in peripheral arterial systems (5–7), little is known about drug behavior in venous tissue or under AVF-specific flow conditions. Furthermore, the relative contributions of tissue type vs. hemodynamic environment remain poorly defined. Addressing this gap is critical for understanding the variable clinical performance of DCBs in AVFs.

In this study, we used a controlled *ex vivo* AVF bioreactor system to isolate the effect of vascular tissue type on acute paclitaxel retention. By maintaining consistent, physiologically relevant flow and pressure conditions, we directly compared drug retention in venous and arterial tissues at 1- and 24 h following DCB treatment. We additionally evaluated coating transfer morphology using scanning electron microscopy (SEM) to provide mechanistic insight into drug deposition patterns.

## 2 Materials and methods

### 2.1 *Ex vivo* AVF bioreactor system

Porcine jugular veins and carotid arteries used in this study were purchased from Animal Biotech Industries, Inc (Doylestown, PA). Excess fat, connective tissue, and fascia were dissected from each vessel upon arrival. The vessels were stored in 15 mL centrifuge tubes at −20°C until use.

The *ex vivo* bioreactor system consisted of a culture medium reservoir, a pulsatile gear pump (Micropump Inc, Vancouver, WA), and a vessel housing chamber (Figure 1), maintained within a CO_2_ incubator at 37°C. Culture medium was low-glucose Dulbecco's Modified Eagle Medium with pyruvate. The vessel housing was a transparent silicone tube with 10% agarose gel surrounding the vessel, which provided extravascular support and enabled measurement of the vessel diameter via ultrasound. Flow rate was measured using an ultrasonic flow meter (Transonic Systems Inc., Ithaca, NY) positioned proximal to the vessel housing. Intraluminal pressure was measured using a catheter pressure transducer (Millar Inc., Houston, TX) inserted into the center of the vessel.

**Figure 1:**
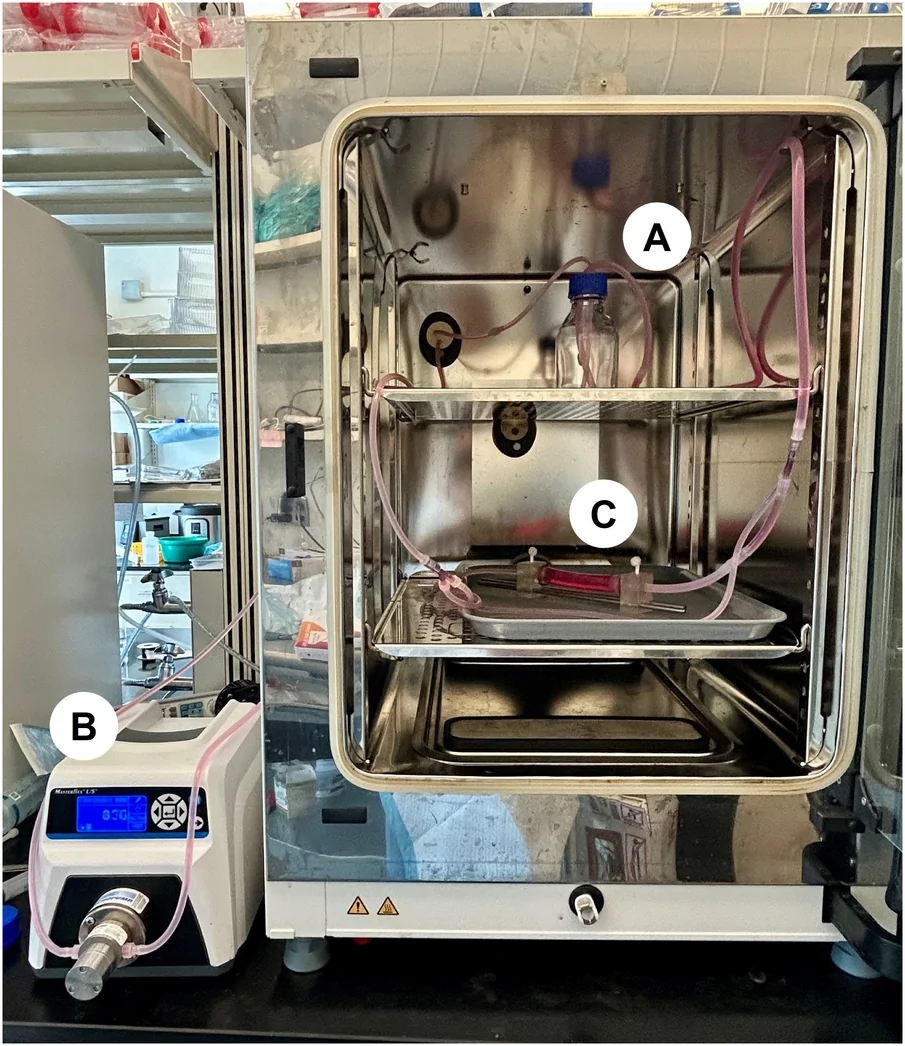
Schematic and image of the *ex vivo* AVF bioreactor system. Culture media is circulated through a closed loop from the **(A)** media reservoir through a pulsatile **(B)** gear pump and into the **(C)** vessel housing. Vessels are embedded in agarose within a compliant silicone tube to provide structural support and enable ultrasound-based diameter measurements.

### 2.2 Drug-coated balloon delivery

The DCB selected for this study was the IN.PACT™ AV (Medtronic Inc, Minneapolis, MN), as this DCB is the most commonly studied device in AVF stenosis clinical trials (4). The DCB was tracked from the proximal end of the vessel with a maximum tracking time of 30 s and inflated to 1.2x the vessel diameter for 2 min based on the device Instructions for Use (8).

### 2.3 Evaluating delivery efficacy

Tissue paclitaxel concentration was measured using a validated, high-performance liquid chromatography (HPLC)-electrospray ionization-tandem mass spectrometry system (LC-MS/MS) assay (9). In brief, treated tissue was homogenized and tissue paclitaxel concentration quantified using a calibration curve of diluted liquid paclitaxel ranging from 0.5 to 100 ng/mL (10, 11). Treated segments were collected at 1 h from the bioreactor system and stored at −80°C until shipped on dry ice to a bioanalytical laboratory (iC42 Clinical Research and Development, Aurora, CO, USA). Tissue paclitaxel concentration is reported in ng of paclitaxel per mg of tissue (ng/mg).

For visualization of drug coating transfer, vessels were collected at 1 h SEM analysis. Samples were cut axially to expose the luminal surface. After drying, samples were sputter-gold coated (LUXOR Goldcoater, Berlin, Germany) and imaged using a Phenom XL scanning electron microscope (Thermo Fisher Scientific, Waltham, MA). Coating deposition was assessed qualitatively from these images.

### 2.4 Statistical analysis

Two-way analysis of variance (ANOVA) was conducted to examine the effect of two independent variables—tissue type (venous vs. arterial tissue) and time point (1 h vs. 24 h)—on tissue paclitaxel concentration. Significance was set at *α* = 0.05. The Shapiro–Wilk test was used to evaluate the normality of the residuals, which revealed significant deviation from normality (*W* = 0.806, *p* < 0.001). Tissue paclitaxel concentrations were therefore log-transformed prior to analysis, after which residuals did not depart from normality (*W* = 0.960, *p* = 0.430). All inferential statistics—ANOVA *F*-tests and 95% confidence intervals (CIs) for between-group differences—are reported on the log-transformed scale. Group statistics are expressed as median and interquartile range (IQR). Between-group differences are expressed as geometric mean ratios (GMR) with 95% CIs back-transformed from the log scale. Where significant main effects were observed, Bonferroni's multiple comparisons *post hoc* test was employed to identify specific group differences. All statistical analyses were conducted using GraphPad Prism 11.

## 3 Results

### 3.1 Hemodynamic characterization of the *ex vivo* AVF benchtop bioreactor model

Consistent and physiologically relevant AVF hemodynamic conditions were achieved across both venous and arterial experimental groups. The average flow rate was 721.06 ± 27.89 mL/min in veins and 666.80 ± 26.69 mL/min in arteries, with no significant difference between groups (*p* = 0.240) (Figure 2). Similarly, systolic and diastolic pressures were comparable, measuring 141.45 ± 9.62/86.53 ± 6.78 mmHg in veins and 157.63 ± 32.35/90.44 ± 17.85 mmHg in arteries (systolic: *p* = 0.371; diastolic: *p* = 0.693) (Figure 3). These results confirm that both vessel types were exposed to similar flow and pressure conditions throughout the experiments.

**Figure 2:**
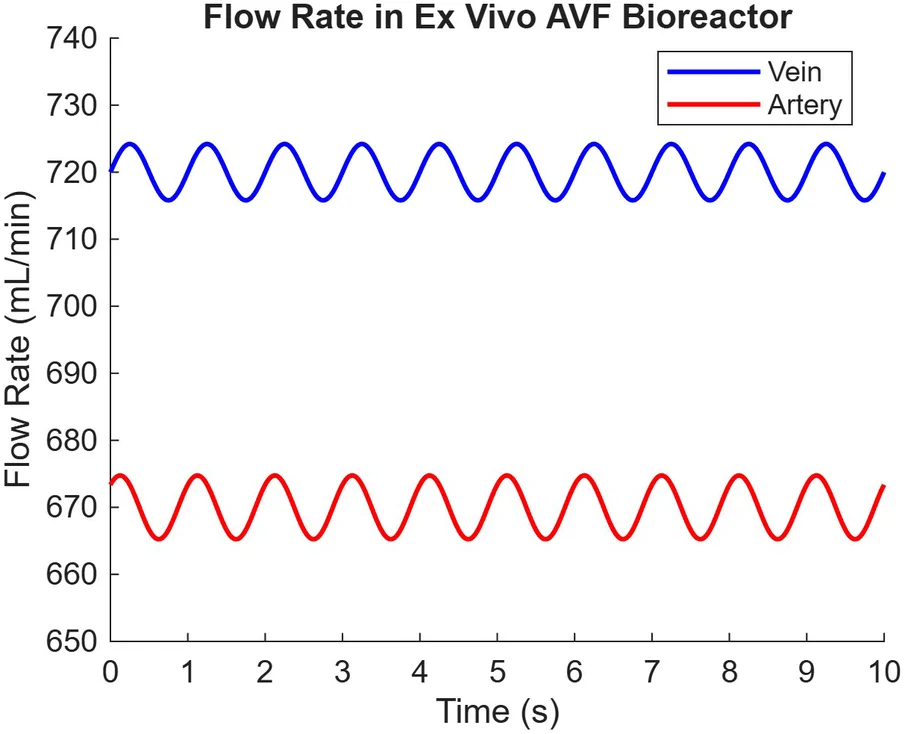
Flow conditions in the *ex vivo* AVF bioreactor system. Comparison of mean volumetric flow rate in the *ex vivo* bioreactor system using venous (blue) and arterial (red) test vessels. Flow rates were not significantly different between groups (*p* = 0.240).

**Figure 3:**
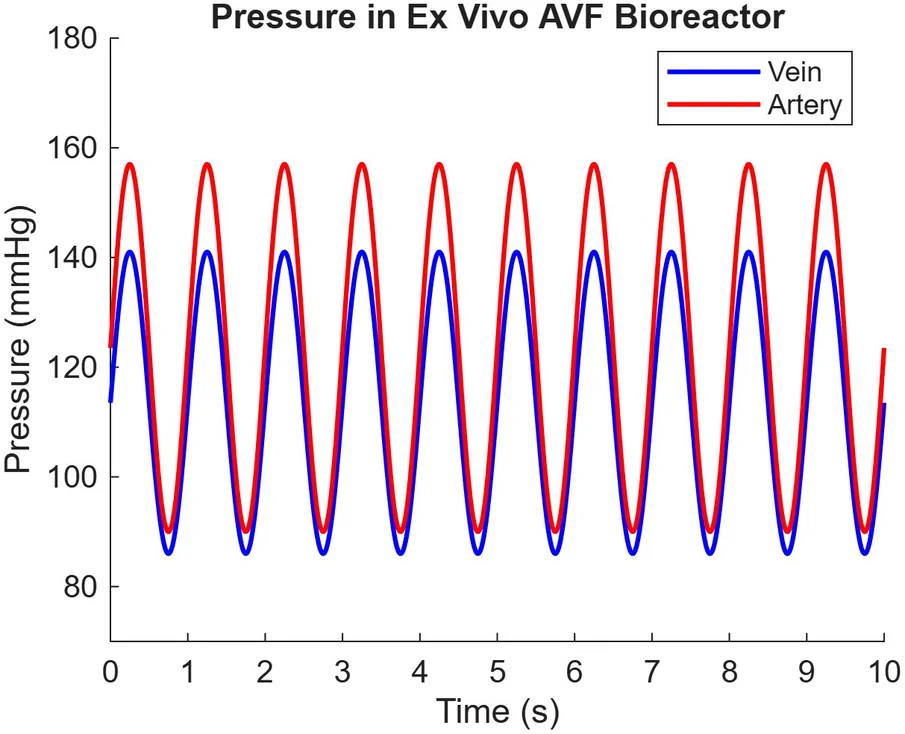
Pressure conditions in the *ex vivo* AVF bioreactor. Comparison of systolic and diastolic pressures in the bioreactor using venous (blue) and arterial (red) test vessels. No significant differences were observed in systolic (*p* = 0.371) or diastolic pressure (*p* = 0.693).

### 3.2 Quantitative assessment of DCB paclitaxel delivery via pharmacokinetic analysis

Paclitaxel was successfully delivered and retained in both venous and arterial tissues at 1- and 24 h following DCB treatment. At 1 h post-treatment, median tissue paclitaxel concentration was 189.0 ng/mg (IQR 52.0–1,882.4 ng/mg; *n* = 9) in venous tissue, compared to 138.3 ng/mg (IQR 101.1–204.7 ng/mg; *n* = 5) in arterial tissue (Figure 4). At 24 h, median concentration had decreased in both tissue types, to 113.8 ng/mg (IQR 61.4–476.1 ng/mg; *n* = 5) in veins and 64.5 ng/mg (IQR 15.6–150.1 ng/mg; *n* = 5) in arteries. Sample sizes ranged from *n* = 5 to 9 per group, as the venous 1 h cohort specifically was expanded during the study after the initial venous samples showed markedly greater spread in tissue paclitaxel concentration than the arterial samples. Therefore, additional venous vessels were run at the 1 h time point to better characterize this variability.

**Figure 4:**
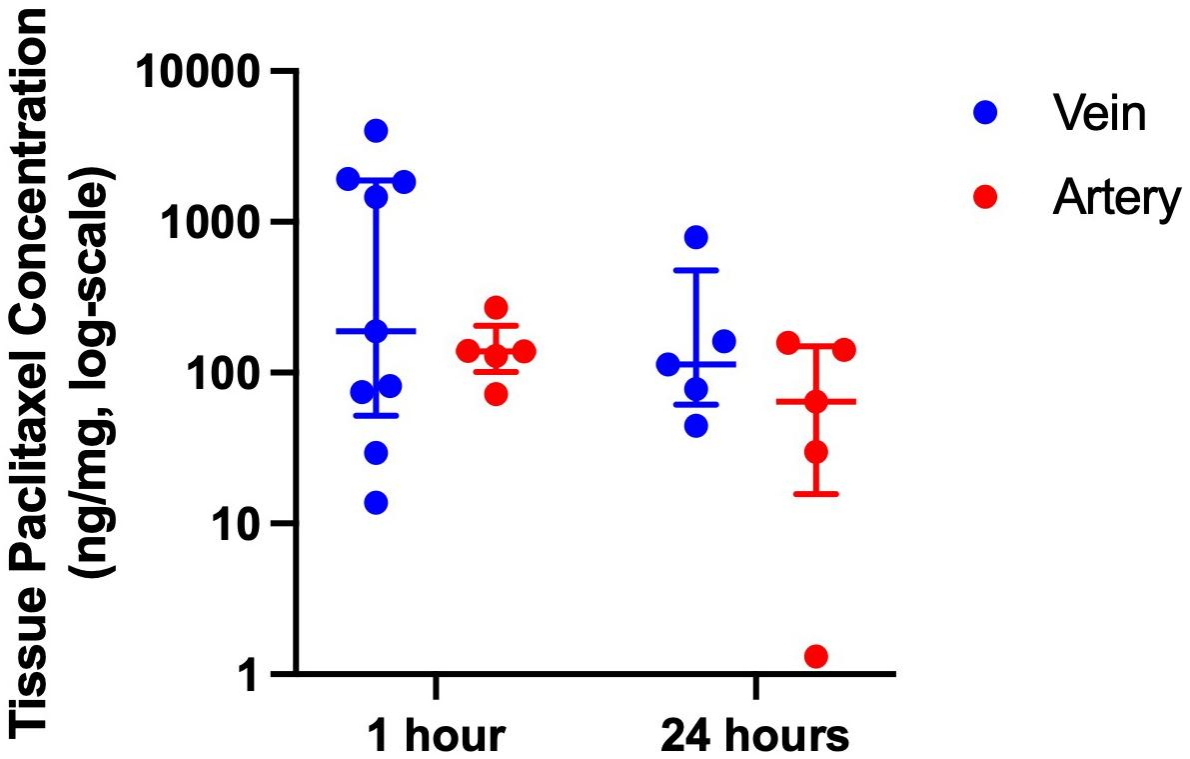
Tissue paclitaxel concentration in veins and arteries treated with iN.PACT™ DCB under AVF flow, grouped by time point (1 h, 24 h). Individual vessel values are plotted on a log scale (vein, blue; artery, red), with horizontal lines indicating the median and interquartile range (IQR) for each group. On log-transformed concentrations, no significant main effects of tissue type (two-way ANOVA, *p* = 0.163) or time (*p* = 0.166) were observed; adjusted geometric mean ratio (vein/artery) = 2.69 (95% CI: 0.65–11.18). *n* = 9 (venous, 1 h), *n* = 5 (venous, 24 h), *n* = 5 (arterial, 1 h), *n* = 5 (arterial, 24 h).

Two-way ANOVA performed on log-transformed concentrations demonstrated no significant main effects of tissue type [F(1,21) = 2.091, *p* = 0.163] or time [F(1,21) = 2.058, *p* = 0.166] on tissue paclitaxel concentration. Back-transformed adjusted GMR of tissue type (vein/artery) was 2.69 [95% CI: 0.65–11.18] and was 2.67 [95% CI: 0.64–11.09] of time (1 h/24 h). These wide CIs reflect the large variability of tissue paclitaxel concentration in venous tissue at 1 h and why no significant differences in tissue paclitaxel concentration were observed between tissue types or timepoints.

### 3.3 Qualitative assessment of DCB coating transfer via scanning electron microscopy

SEM performed at 1 h confirmed successful transfer of the drug coating to the endothelial surface of both venous and arterial tissues (Figure 5). In both vessel types, coating deposition was heterogeneous and non-uniform.

**Figure 5:**
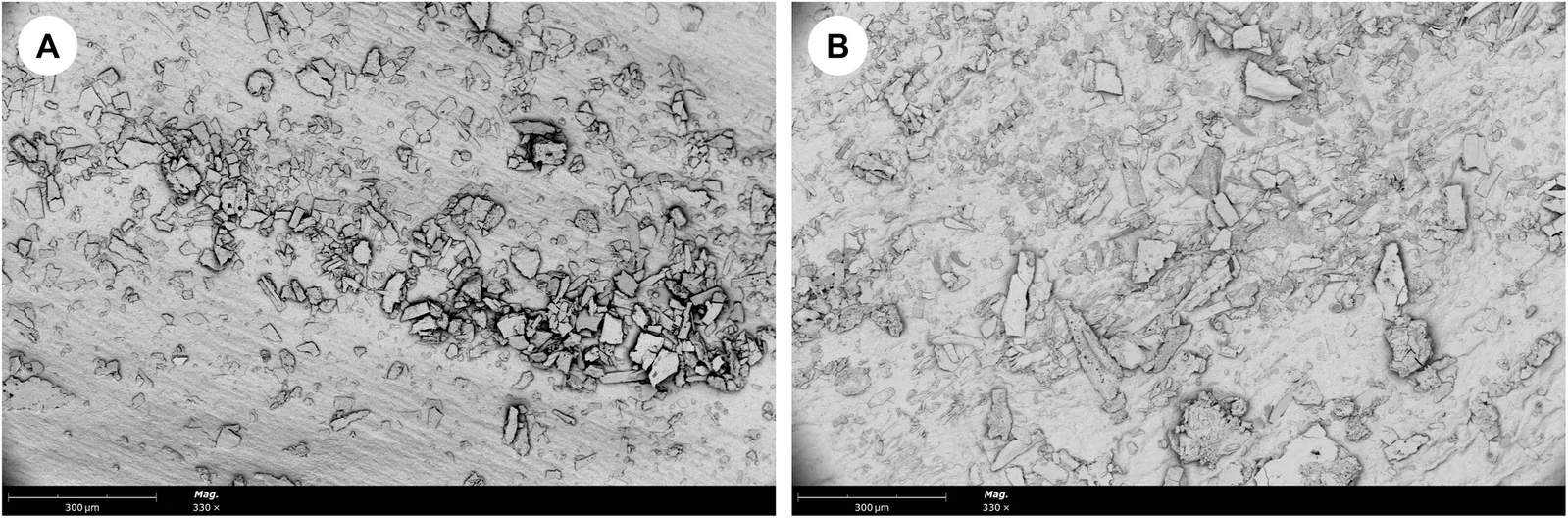
Morphology of DCB coating transfer to vascular endothelium. Representative SEM images of paclitaxel coating deposited on the luminal surface of **(A)** venous and **(B)** arterial tissue 1 h following DCB treatment under AVF flow. Both tissue types exhibit heterogeneous, non-uniform coating transfer. Venous samples show larger, clustered aggregates of coating material, whereas arterial samples display more distributed but discontinuous surface coverage. Scale bars: 300 μm.

Qualitative differences in the deposition patterns were observed between tissue types. Venous samples exhibited larger, clustered aggregates of coating material, whereas arterial samples showed relatively more evenly distributed, though still discontinuous, surface coverage. Despite these differences in spatial distribution, both tissue types demonstrated incomplete and irregular coating retention.

## 4 Discussion

The first clinical use of DCBs in vascular access in 2012 was inspired by their demonstrated ability to inhibit neointimal hyperplasia and reduce restenosis in the superficial femoral artery (12). However, despite more than a decade of subsequent clinical trials, DCBs have consistently shown only short- to medium-term advantages over plain balloon angioplasty in AVFs, with long-term superiority remaining uncertain (13). Despite extensive clinical evaluation, the factors underlying the variable performance of DCBs in AVFs remain poorly understood, particularly with respect to how vascular tissue type and hemodynamic environment influence drug delivery and retention.

The delivery mechanism of DCBs relies on efficient transfer of the drug coating from the balloon catheter to the tissue. This coating is composed of an antiproliferative drug and an excipient, which, in the case of IN.PACT™ AV, are paclitaxel and urea, respectively. The lipophilicity of paclitaxel promotes rapid uptake into vascular tissue and supports sustained retention, with measurable levels persisting for weeks after a single administration (14). The hydrophilicity of urea enhances coating adhesion and drug solubility to facilitate transfer of the paclitaxel into the vessel wall during balloon-tissue contact. This urea-based coating has a rapid integration of the solid phase and a slow and relatively linear rate of dissolution, approximately 0.3% of transferred drug load/hour in arterial tissue (7). Drug retention is, therefore, shaped by both paclitaxel binding within the tissue and physical coating transfer to the vessel surface. This emphasizes the importance of DCB coating development to consider maximizing bulk transfer and adhesion for a 2–3 min delivery period and balancing solid phase dissolution with tissue clearance of soluble drug.

Experimental platforms are well suited to examine these factors because they enable controlled assessment of tissue- and hemodynamic-dependent variables while minimizing confounding influences such as immune responses. Existing benchtop AVF models primarily utilize compliant silicone tubes (15) or 3D-printed, patient-specific anastomosis geometries (16) to investigate systemic and local hemodynamic effects of AVF configuration (17, 18). While these systems provide valuable insight into flow dynamics, they do not enable direct assessment of drug transport and tissue retention. In contrast, previous studies by Cooper et al. and Chen et al. describe *ex vivo* peripheral arterial bioreactor systems that incorporate biological tissue and support evaluation of acute pharmacokinetics of drug-eluting devices (9, 19).

Building on these approaches, we developed a novel *ex vivo* AVF bioreactor system that integrates biological tissue and reproduces clinically relevant AVF flow and pressure conditions. In this study, we used this platform to perform the first head-to-head comparison of DCB performance in venous vs. arterial tissue under controlled AVF hemodynamics. Reported AVF flow rates and pressures (∼750 mL/min and 145/90 mmHg) (20) were closely matched in our system, confirming physiological relevance. Under these controlled conditions, we found that paclitaxel retention did not differ significantly between venous and arterial tissues. This estimate, however, was accompanied by a wide 95% confidence interval (adjusted GMR [vein/artery] = 2.69, 95% CI: 0.65–11.18), reflecting limited precision in the between-group comparison. Because flow rate and pressure were maintained consistently across experiments, both vessel types were exposed to comparable mechanical conditions; thus, the absence of differences in drug retention is unlikely to be attributable to variations in hemodynamic exposure.

This absence of a statistically significant difference in drug retention between veins and arteries is somewhat unexpected given their distinct biological properties and the delivery mechanism of DCBs. Veins are generally more permeable due to less restrictive endothelial junctions (21), which could facilitate drug entry into the vessel wall. Conversely, paclitaxel's strong lipophilicity favors strong partitioning into tissue (22). Complicating these opposing effects, elastin, which is particularly abundant in arteries, can hinder paclitaxel diffusion (23). These competing mechanisms of enhanced permeability in veins vs. increased binding and structural resistance in arteries may offset one another and could contribute to the lack of a statistically significant difference in net drug retention observed under high-flow AVF conditions. This interpretation is further supported by the similar reduction in drug concentration observed in both tissue types from 1- to 24 h, suggesting that washout or redistribution processes are not strongly dependent on vessel type within this acute time frame.

Although no statistically significant differences were observed, venous samples demonstrated substantially greater variability in paclitaxel concentration, particularly at the 1 h time point. This variability may have contributed to the absence of detectable differences despite higher mean values in the venous group. One potential source of this variability is the presence of venous valves, which were not controlled for relative to the site of drug delivery. Valve leaflets, composed largely of collagen and elastin (24), may alter local drug deposition and retention by creating regions of altered surface geometry and drug binding capacity. As a result, drug delivery to or near valve structures could introduce variability in measured tissue concentrations. These findings highlight an important and clinically relevant source of variability in AVF interventions, where anatomical features may influence treatment efficacy.

Morphological analysis using SEM provided additional insight into differences in coating transfer between tissue types. While both veins and arteries exhibited successful yet non-uniform drug deposition, the spatial distribution of coating differed qualitatively. Venous samples displayed larger, clustered aggregates of coating material, whereas arterial samples showed more dispersed but still discontinuous coverage. These observations are consistent with differences in vessel compliance, which can influence balloon–vessel wall contact pressure, a factor known to affect DCB coating transfer (25). Veins are up to 30 times more compliant than arteries (26) and may experience reduced balloon–vessel wall contact pressure during inflation, leading to uneven transfer and localized accumulation of coating. In contrast, the stiffer arterial wall may support more uniform contact, resulting in broader distribution of drug on the luminal surface. Despite these differences in deposition patterns, the lack of statistical differences in retained drug concentration suggests that variation in initial coating transfer does not necessarily translate into differences in bulk tissue drug levels.

Several limitations should be considered when interpreting this study. First, healthy native porcine vessels were used rather than diseased or arterialized veins. This is consistent with the current FDA preclinical testing standards which utilize healthy animal models (27). However, pathological features of diseased AVF tissue—such as neointimal hyperplasia, fibrosis, chronic inflammation, and altered extracellular matrix composition that characterize diseased, stenotic AVF segments in clinical practice—could substantially influence drug uptake and retention. Therefore, absolute retention values reported here should not be directly extrapolated to diseased AVF tissue, and future development of *ex vivo* AVF models beyond the exploratory model described in this study would benefit from inclusion of diseased tissue. Second, while the bioreactor system reproduces key aspects of hemodynamics in the outflow vessels of the AVF circuit, it does not reflect the anatomy of the AVF anastomosis. This complex geometry has been shown to significantly impact the local hemodynamics of the AVF (28), and therefore, may not reflect the oscillatory shear and flow disturbance of the anastomosis. Third, the small sample size used in this study limits the statistical power of the analysis. Relatedly, this study was not designed or powered as a formal equivalence or non-inferiority trial, and no predefined equivalence margin for *ex vivo* tissue paclitaxel retention currently exists in the literature. Furthermore, variability in the venous data, while informative, also limits statistical power and highlights the need for tighter control of anatomical features such as valve location in future studies. Finally, assessment of DCB coating transfer by SEM was qualitative, as quantitative morphometric analysis of coating deposition (e.g., percentage of luminal surface coverage, particle or aggregate size, particle density) was beyond the scope of this study.

In summary, this study provides the first direct comparison of DCB delivery between venous and arterial tissue under controlled, physiologically relevant AVF conditions. Despite notable differences in structure, compliance, and coating deposition patterns, venous and arterial tissues showed no statistically significant difference in acute paclitaxel retention under AVF hemodynamics. These findings suggest that tissue type alone may not be the primary determinant in early DCB drug retention. Instead, other factors such as lesion-specific pathology, vascular remodeling, inflammation, and long-term biological responses likely play a more significant role in the inconsistent clinical performance of DCBs in AVFs compared to their well-established efficacy in peripheral arterial disease. This work further underscores the importance of considering the complex interaction between device, tissue, and hemodynamic environment when evaluating DCB therapies for AVF applications and establishes an *ex vivo* bioreactor model as a valuable platform for evaluating catheter-based drug delivery systems in AVF-specific conditions.

## Data Availability

The original contributions presented in the study are included in the article/Supplementary Material, further inquiries can be directed to the corresponding author/s.
